# Supplementary material for: Evaluation of Five Buffers for Inactivation of Monkeypox Virus and Feasibility of Virus Detection Using the Panther Fusion® Open Access System
Source: Viruses. 2022 Oct 10;14(10):2227. doi: 10.3390/v14102227 (PMC9610623; doi:10.3390/v14102227)
Supplement: Supplementary file 1 [file viruses-1960626-supplementary.pdf › viruses-1960626-supplementary.pdf]

Table S1. Reagent removal methods that were inconclusive. Cells in all of the wells including the no virus controls were missing or extremely damaged.

Reagent	Time	Reagent Removal Method	Plaque Count								
			Sample 1			Sample 2			Sample 3		
			Well 1	Well 2	Well 3	Well 1	Well 2	Well 3	Well 1	Well 2	Well 3
Panther STM	10	de-salt	inconclusive - cells lifted from plate								
Panther UTM	10	detergent	inconclusive - cells lifted from plate								
		detergent X 2	inconclusive - cells lifted from plate								
		Amicon	inconclusive - cells lifted from plate								
Trizol	10	detergent	inconclusive - cells lifted from plate								
AVL & Ethanol	10	de-salt	inconclusive - cells lifted from plate								
